# The small GTPase ARF6 regulates GABAergic synapse development

**DOI:** 10.1186/s13041-019-0543-3

**Published:** 2020-01-06

**Authors:** Hyeonho Kim, Hyeji Jung, Hyunsu Jung, Seok-Kyu Kwon, Jaewon Ko, Ji Won Um

**Affiliations:** 1 Department of Brain and Cognitive Sciences, Daegu Gyeongbuk Institute of Science and Technology (DGIST), 333 Techno Jungangdae-Ro, Hyeonpoong-eup, Dalseong-gun, Daegu, 42988 South Korea; 2 Division of Life Sciences, Korea University, Seoul, 02841 South Korea; 3 Center for Functional Connectomics, Brain Science Institute, Korea Institute of Science and Technology, Seoul, 02792 South Korea

**Keywords:** ARF, Epilepsy, GABA, Inhibitory synapse

## Abstract

ADP ribosylation factors (ARFs) are a family of small GTPases composed of six members (ARF1–6) that control various cellular functions, including membrane trafficking and actin cytoskeletal rearrangement, in eukaryotic cells. Among them, ARF1 and ARF6 are the most studied in neurons, particularly at glutamatergic synapses, but their roles at GABAergic synapses have not been investigated. Here, we show that a subset of ARF6 protein is localized at GABAergic synapses in cultured hippocampal neurons. In addition, we found that knockdown (KD) of ARF6, but not ARF1, triggered a reduction in the number of GABAergic synaptic puncta in mature cultured neurons in an ARF activity-dependent manner. ARF6 KD also reduced GABAergic synaptic density in the mouse hippocampal dentate gyrus (DG) region. Furthermore, ARF6 KD in the DG increased seizure susceptibility in an induced epilepsy model. Viewed together, our results suggest that modulating ARF6 and its regulators could be a therapeutic strategy against brain pathologies involving hippocampal network dysfunction, such as epilepsy.

## Introduction

ADP-ribosylation factor 6 (ARF6) belongs to the ARF protein family of small GTPases known to regulate actin remodeling and membrane trafficking [1]. Like other small GTPases, ARFs function as molecular switches by cycling active GTP-bound and inactive GDP-bound forms, a process that is tightly regulated by guanine nucleotide exchange factors (GEFs) and GTPase-activating proteins (GAPs) [2]. Functionally, ARF1 and ARF6 have been the most extensively studied in neurons; ARF1 is essential for regulating transport between intra-Golgi compartments, whereas ARF6 regulates the recycling of endosomes and receptors to and from the plasma membranes, and modulates cortical cytoskeletal organization [1]. In particular, the roles of ARF6 at excitatory synapses have been well described. For example, ARF6 promotes the conversion of immature filopodia to mature dendritic spines, and enhances the stability of early spines in cultured hippocampal neurons by regulating dendritic development, and axonal elongation and branching in postsynaptic neurons during neuronal development [3–6]. ARF6 also controls the endocytosis of synaptic vesicles in presynaptic neurons [7]. Moreover, loss of ARF6 function induces activity-dependent accumulation of endosomal structures and increases release-competent docked synaptic vesicles, suggesting an active role of ARF6 in regulating cycling and synaptic vesicle pools at presynaptic neurons [8].

Similarly, synaptic roles of several GEFs at synapses have been identified and investigated. The ARF-specific GEF, msec7–1 (a rat homologue of human cytohesin-1/ARNO), directly interacts with Munc13–1 at presynaptic active zones to regulate presynaptic cycling of synaptic vesicles [9, 10]. In addition, overexpression of msec7–1 in *Aplysia* neurons increases the number of neuronal processes and varicosities along neurites in an ARF-GEF activity-dependent manner, suggesting a role for msec7–1 in neuritogenesis [11]. EFA6A, an ARF6-specific GEF, is highly expressed in brains and is critical for dendritic spine development and maintenance [3, 12]. Deletion of another EFA6 isoform, EFA6C/Psd2, in mice reduces synaptic density in Purkinje neurons of the cerebellum [13]. Still another ARF6-specific GEF, BRAG1 (synonymous with IQSEC2 [14];), interacts with PSD-95 and some PDZ domain-containing scaffolds through its C-terminal PDZ domain-binding sequence and binds to IRSp53 (also known as BAIAP2) through its proline-rich sequence to form multiprotein complexes at excitatory synapses of postsynaptic neurons [15–17]. BRAG1/IQSEC2 also regulates AMPA receptor trafficking to modulate long-term synaptic depression (LTD) [18], and mediates ARF6 activation in regulating AMPA receptor trafficking and LTD [19]. TBC1 domain family member 24 (TBC1D24) interacts with ARF6 and regulates neuronal migration and dendritic outgrowth by preventing ARF6 activation [20]. Synaptic roles of a subset of ARF GAPs have also been revealed. In particular, G-protein-coupled receptor kinase-interacting protein 1 (GIT1; an ARF6 GAP) has been extensively studied. GIT1 regulates neurotransmitter release probability and vesicle recycling at presynaptic neurons [21, 22] and modulates AMPA receptor targeting and dendritic spine morphogenesis at postsynaptic neurons [23, 24]. Similarly, AGAP1 regulates actin cytoskeleton and dendritic spine morphology [25, 26].

Despite these overarching studies, the roles of ARF6 at GABAergic synapses are relatively poorly understood. However, it is possible to propose that normal ARF6 function is crucial for GABAergic synapse development, as evidenced by reported actions of ARF6 GEFs and GAPs at GABAergic synapses. GIT1 regulates GABA_A_R trafficking and GABAergic synaptic transmission [27], whereas IQSEC3/BRAG3 directly interacts with gephyrin to regulate GABAergic synapse formation [17, 28–30].

In the present study, we showed that ARF6 activity is critical for GABAergic synapse development and hippocampal network activity. ARF6 knockdown (KD) in cultured hippocampal neurons decreased GABAergic synapse density, an effect that was completely rescued by ARF6 wild-type (WT) and ARF6-T157A (a fast cycling mutant), but not by ARF6-T27 N (a dominant-negative mutant). In addition, ARF6 KD in the mouse hippocampal DG area reduced GABAergic synapse density, which in turn affected the activity of neuronal populations in the mouse hippocampus and increased susceptibility to kainic acid (KA)-induced seizures.

## Materials and methods

### Construction of expression vectors

Small hairpin RNA (shRNA) lentiviral expression vectors against *Arf6* and *Arf1* were constructed by annealing, phosphorylating, and cloning oligonucleotides targeting rat *Arf6* (5′-AGCTGCACCGCATTATCAA-3′) or *Arf1* (5′-ACTGTTGAATACAAGAATA-3′) into *Xho*I and *Xba*I sites of a single KD vector (L-315) [3, 31]. For the ARF6 rescue vector, three nucleotides (underlined) in the AGCTGCACGCATTATCAA sequence of GW1-HA-ARF6 were mutated to render them shRNA-resistant. The shRNA AAV against mouse *Arf6* (Genbank accession number: NM_007481.3) was constructed by annealing, phosphorylating, and cloning oligonucleotides targeting mouse *Arf6* (5′-AGCTGCACCGCATTATCAA-3′) into *BamH*I and *EcoR*I sites of the pAAV-U6-GFP vector (Cell BioLabs, Inc.). AAVs encoding full-length human ARF6 WT and ARF6-T27 N, and ARF6-T157A point mutants were generated by amplification of full-length ARF6 by polymerase chain reaction (PCR) and subsequent subcloning into the pAAV-2A-EGFP vector (a gift from Hailan Hu [32];) at *Xba*I and *BamH*I sites. cDNA encoding full-length human ARF6 WT, ARF6-T27 N, and ARF6-T157A point mutants were amplified by PCR and subcloned into the L-313 vector (see [33]), using *BamH*I and *EcoR*I sites. pCAG-gephyrin-tdTomato was kindly gifted from Drs. Franck Polluex and Daniel Isacone (Columbia University).

### Neuron culture, transfections, imaging, and quantitation

Cultured hippocampal rat neurons were prepared from embryonic day 18 (E18) rat embryos, as previously described [34], cultured on coverslips coated with poly-D-lysine (Sigma), and grown in Neurobasal medium supplemented with B-27 (Thermo Fisher), 0.5% fetal bovine serum (FBS; WELGENE), 0.5 mM GlutaMax (Thermo Fisher), and sodium pyruvate (Thermo Fisher). For knockdown of ARF1 or ARF6 in cultured neurons, hippocampal neurons were transfected with L-315 alone (Control), L-315 sh-Arf1 or L-315 sh-Arf6, or cotransfected with ARF6-KD and shRNA-resistant HA-ARF6 using a CalPhos Kit (Clontech) at 8 days in vitro (DIV8) and immunostained at DIV14. For *ex utero* electroporation experiments, lateral brain ventricles of embryos isolated from timed-pregnant ICR mice (E15.5) were injected with a plasmid (2 μg/μl) and 0.5% Fast Green (Sigma) mixture and electroporated with four pulses of 20 V for 100 ms at 500-ms intervals using an ECM830 electroporation system. Electroporated embryonic cortices were dissected and isolated in Hank’s Balanced Salt Solution (HBSS) containing 10 mM HEPES (pH 7.4), and incubated in HBSS containing 14 U/ml papain (Worthington) and 100 μg/μl DNase I for 15 min at 37 °C. After washing, tissues were dissociated by pipetting, and plated on poly-D-lysine and laminin-coated coverslips (Corning) in Neurobasal media (Invitrogen) supplemented with B27 (Invitrogen), Glutamax (Invitrogen), 2.5% FBS (Invitrogen), and 0.5x penicillin/streptomycin (Invitrogen). After 1 week, half of the medium was replaced with FBS-free medium. For immunocytochemistry, cultured neurons were fixed with 4% paraformaldehyde/4% sucrose, permeabilized with 0.2% Triton X-100 in phosphate-buffered saline (PBS), immunostained with the indicated primary antibodies, and detected with Cy3- and fluorescein isothiocyanate (FITC)-conjugated secondary antibodies (Jackson ImmunoResearch). Images were acquired using a confocal microscope (LSM700, Carl Zeiss) with a 63x objective lenses; all image settings were kept constant. Z-stack images were converted to maximal projection and analyzed to obtain the size, intensity, and density of immunoreactive puncta derived from marker proteins. Quantification was performed in a blinded manner using MetaMorph software (Molecular Devices).

### Antibodies

The following commercially available antibodies were used: goat polyclonal anti-EGFP (Rockland), chicken polyclonal anti-EGFP (Aves Labs), rabbit polyclonal anti-RFP (Abcam), mouse monoclonal anti-HA (clone 16B12; Covance), mouse monoclonal anti-GAD67 (clone 1G10.2; Millipore), guinea pig polyclonal anti-VGLUT1 (Millipore), mouse monoclonal anti-gephyrin (clone 3B11; Synaptic Systems), rabbit polyclonal anti-VGAT (Synaptic Systems), rabbit polyclonal anti-GABA_A_Rγ2 (Synaptic Systems), mouse monoclonal anti-PSD-95 (clone K28/43; Neuromab), mouse monoclonal anti-gephyrin (clone 3B11; Synaptic Systems), and rabbit polyclonal anti-ARF6 (Abcam). Rabbit polyclonal anti-IQSEC3 (JK079) [29] and guinea pig polyclonal anti-IQSEC3/SynArfGEF (a gift from Dr. Hiroyuki Sakagami) [35] antibodies were previously described.

### Production of recombinant viruses

#### AAVs

Recombinant AAVs were packaged with pHelper and AAV1.0 (serotype 2/9) capsids for high efficiency. HEK293T cells were cotransfected with pHelper and pAAV1.0, together with pAAV-U6-EGFP alone (Control), pAAV-U6-shArf6 (ARF6 KD), pAAV-ARF6 WT-2A-EGFP (ARF6-WT), pAAV-ARF6-T27 N-2A-EGFP (ARF6-T27 N), or pAAV-ARF6-T157A-2A-EGFP (ARF6-T157A). Transfected HEK293T cells were harvested 72–108 h post transfection. After addition of 0.5 M EDTA to the medium, cells were washed three times with PBS and collected by centrifugation. Cells were then resuspended in PBS and lysed by subjecting to four freeze-thaw cycles in an ethanol/dry ice bath (7 min each) and 37 °C water bath (5 min each). Lysates were centrifuged, and supernatants were collected and incubated with a solution containing 40% poly (ethylene glycol) (Sigma) and 2.5 M NaCl on ice for 1 h, and centrifuged at 2000 rcf for 30 min. The pellets were resuspended in HEPES buffer (20 mM HEPES pH 8.0, 115 mM NaCl, 1.2 mM CaCl_2_, 1.2 mM MgCl_2_, 2.4 mM KH_2_PO_4_), mixed with chloroform, and centrifuged at 400 rcf for 10 min. The supernatant was collected and concentrated using Amicon Ultra Centrifugal Filters (0.5 ml, 3 K MWCO; Millipore). Viruses were assessed for infectious titer by RT-PCR, and used for infections at 1 × 10^10^–10^12^ infectious units/μl.

#### Lentiviruses

Lentiviruses were produced by transfecting HEK293T cells with L-315 empty vector or L-315-sh-Arf6, with packaging vectors (pMD2G and psPAX), as previously described [33].

### Animals and ethics statement

C57BL/6 N mice (purchased from Jackson Laboratory, ME, USA; stock number: 013044) were maintained and handled in accordance with protocols approved by the Institutional Animal Care and Use Committee of DGIST under standard, temperature-controlled laboratory conditions. Mice were maintained on a 12:12 light/dark cycle (lights on at 7:00 am and off at 7:00 pm), and received water and food ad libitum. All experimental procedures were performed on male mice. Pregnant rats purchased from Daehan Biolink were used for in vitro culture of dissociated cortical or hippocampal neurons. All procedures were conducted according to the guidelines and protocols for rodent experimentation approved by the Institutional Animal Care and Use Committee of DGIST.

### Stereotaxic surgery and virus injections

For stereotaxic delivery of recombinant AAVs, 9–week-old C57BL/6 N mice were anesthetized by inhalation of isoflurane (3–4%) or intraperitoneal injection of a saline solution containing 2% 2,2,2-tribromoethanol (Sigma), and secured in a stereotaxic apparatus. Viral solutions were injected with a Hamilton syringe using a Nanoliter 2010 Injector (World Precision Instruments) at a flow rate of 100 nl/min (injected volume, 0.6 μl). The coordinates used for stereotaxic injections into the hippocampal DG of mice were as follows: anteroposterior (AP), − 2.2 mm; medial–lateral (ML), ± 1.3 mm; and dorsal–ventral (DV), 2.2 mm from bregma. Each injected mouse was returned to its home cage and after 2 weeks was used for scoring seizure-like behaviors, immunohistochemical analyses, or electrophysiological recordings.

### Immunoblot analysis of infected brain tissues

Brain regions infected with the indicated AAVs were homogenized in 0.32 M sucrose/1 mM MgCl_2_ containing a protease inhibitor cocktail (Thermo-Fisher Scientific) using a Precellys Evolution tissue homogenizer (Bertin Co.). After centrifuging homogenates at 1000×g for 10 min, the supernatant was transferred to a fresh microcentrifuge tube and centrifuged at 15,000×g for 30 min. The resulting synaptosome-enriched pellet (P2) was resuspended in lysis buffer and centrifuged at 20,800×g, after which the supernatant was analyzed by Western blotting with anti-ARF6 antibodies.

### Seizure behavior scoring

Nine-week-old male C57BL/6 N mice stereotactically injected with the indicated AAVs were administered KA (20 mg/kg; Sigma Cat. No. K0250) or saline (control), and the resulting seizure behaviors were video-recorded for the next 2 h. Seizure susceptibility was measured by rating seizures every 3 min on a scale of 0 to 5 as follows: 0, no abnormal behavior; 1, reduced motility and prostate position; 2, partial clonus; 3, generalized clonus including extremities; 4, tonic-clonic seizure with rigid paw extension; and 5, death.

### Data analysis and statistics

All data are expressed as means ± SEM. All experiments were repeated using at least three independent cultures, and data were statistically evaluated using a Mann-Whitney U test, analysis of variance (ANOVA) followed by Tukey’s post hoc test, Kruskal-Wallis test (one-way ANOVA on ranks) followed by Dunn’s pairwise post hoc test, or paired two-tailed t-test, as appropriate. Prism7.0 (GraphPad Software) was used for analysis of data and preparation of bar graphs. *P-*values < 0.05 were considered statistically significant (individual *p*-values are presented in figure legends).

## Results

### ARF6 is localized at both GABAergic synapses and glutamatergic synapses in cultured hippocampal neurons

**Fig. 1:**
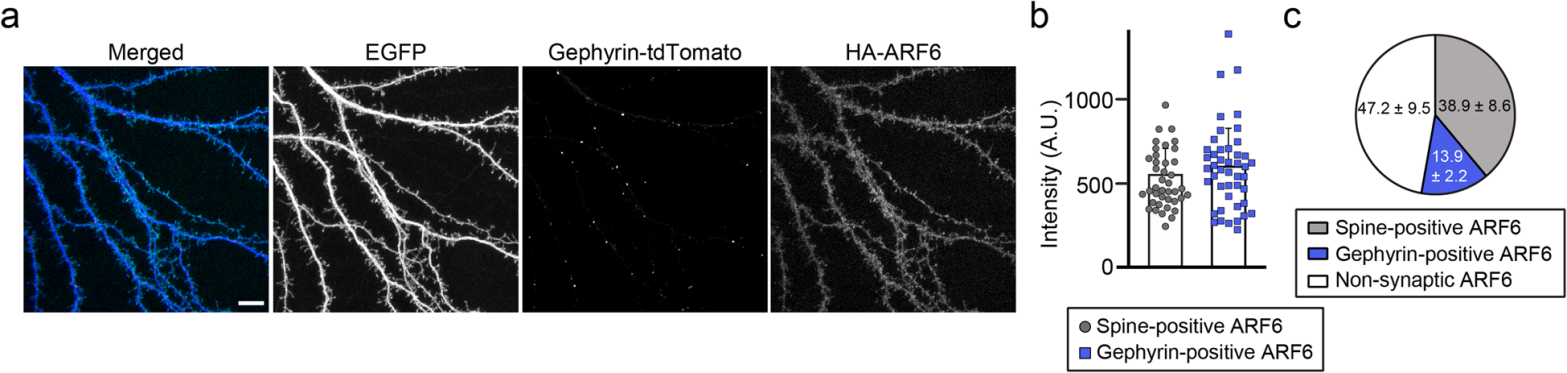
ARF6 is localized to GABAergic synapses. **a**, Representative images of cultured mouse cortical neurons from mouse embryos electroporated at E15.5 with Arf6-HA-IRES-EGFP and gephyrin-tdTomato. Cultured cortical neurons were subsequently immunostained for HA at DIV14. Scale bars, 10 μm. **b** Summary data showing the average intensity of ARF6 at the dendritic spine and gephyrin^+^ puncta. Data are presented as means ± SEMs (*n* = 40–45 ARF6^+^ immunoreactive puncta). **c** Pie chart showing the proportion of HA-ARF6 immunoreactive signals at dendritic spines, gephyrin-positive inhibitory synapses, and non-synaptic sites (spine-negative and gephyrin-negative immunoreactive puncta)

Our previous study demonstrating that the ARF-GEF activity of IQSEC3 is required for maintenance of GABAergic synapse structure raised the possibility that normal levels of ARF activity are crucial for GABAergic synapse development. To date, however, the precise localization of native ARF proteins in neurons has remained unclear, and only a few ARF regulators (i.e., GEFs and GAPs) have been reported to localize to GABAergic synaptic sites. To address the role of ARF6 proteins in mediating GABAergic synapse development, we first performed an immunofluorescence analyses of the synaptic localization of ARF6 in cultured cortical neurons (DIV14) *ex utero* electroporated with ARF6-HA-IRES-EGFP and gephyrin-tdTomato at E15.5 (our ARF6 antibody was not suitable for immunocytochemical applications in brain sections) (Fig. 1a-c). We found that a subset of ARF6-HA immunoreactive signals colocalized with gephyrin-tdTomato puncta (13.9 ± 2.2%), whereas majority of ARF6-HA signals localized to excitatory synaptic spines (38.9 ± 8.6%) or non-synaptic sites (47.2 ± 9.5%), suggesting that a fraction of ARF6 proteins is localized to GABAergic synapses (Fig. 1a–c).

### Knockdown of ARF6 decreases inhibitory synaptic density in cultured neurons

**Fig. 2:**
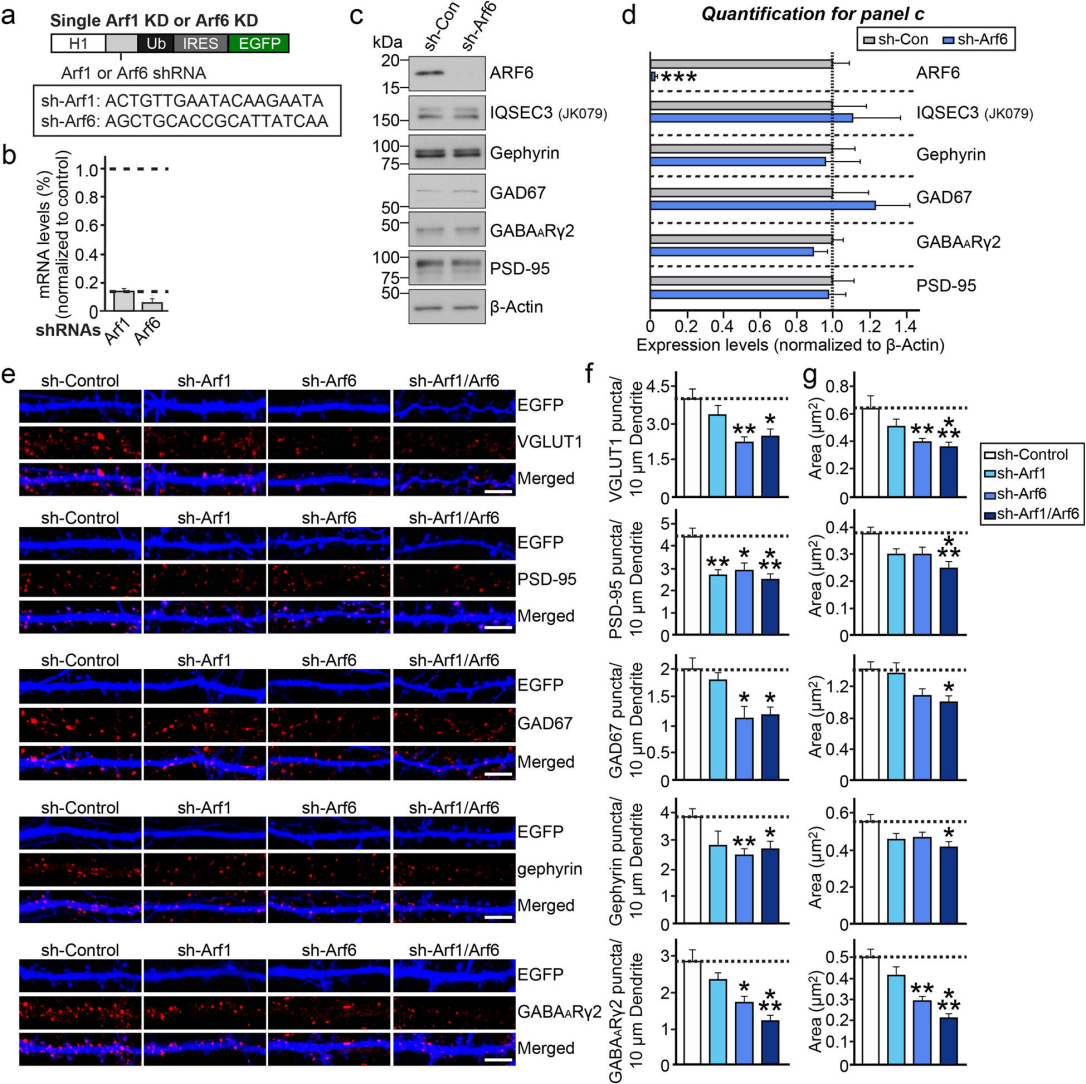
Effects of ARF1 or ARF6 KD on synaptic structures in cultured hippocampal neurons. **a** Design of lentiviral shRNA vectors for KD of ARF1 or ARF6. Boxes indicate shRNA target sequences in *Arf1* and *Arf6*. Abbreviations: H1, human H1 promoter; IRES, internal ribosome entry sequence; Ub, ubiquitin promoter. **b** *Arf1* and *Arf6* mRNA levels in cultured cortical neurons infected at DIV3 with lentiviruses expressing sh-Arf1 or sh-Arf6 were measured by qRT-PCR. mRNA was prepared at DIV10. Dashed line, 85% KD cutoff level for tests of biological effects. Data are presented as means ± SEMs (*n* = 3 independent experiments; *p < 0.05 vs. control; Mann-Whitney U test). **c** Cultured cortical neurons were infected with lentiviruses expressing sh-Arf6 at DIV3 and then immunoblotted with the indicated antibodies at DIV10. **d** Quantification of ARF6, IQSEC3, gephyrin, and PSD-95 levels from **c**, normalized to control. Data are presented as means ± SEMs of three experiments (***p < 0.001 vs. control; Mann-Whitney U test). **e** Representative images of cultured hippocampal neurons transfected at DIV8 with lentiviral constructs expressing EGFP alone (Control), sh-Arf1, sh-Arf6 or cotransfected with sh-Arf1 and sh-Arf6 (sh-Arf1/Arf6). Neurons were analyzed by double-immunofluorescence labeling for EGFP (blue; pseudo-colored) and VGLUT1, PSD-95, GAD67, gephyrin or GABA_A_Rγ2 (red) at DIV14. Scale bar, 10 μm (applies to all images). **f**, **g** Summary data showing the effects of ARF1 KD, ARF6 KD or ARF1 and ARF6 DKD (double-knockdown) in neurons on synaptic puncta density (**f**) and synaptic puncta size (**g**). Data are presented as means ± SEMs (2–3 dendrites per transfected neurons were analyzed and group-averaged; *n* = 22–30 neurons; *p < 0.05, **p < 0.01, ***p < 0.001 vs. control; non-parametric ANOVA with Kruskal-Wallis test followed by post hoc Dunn’s multiple comparison test)

**Fig. 3:**
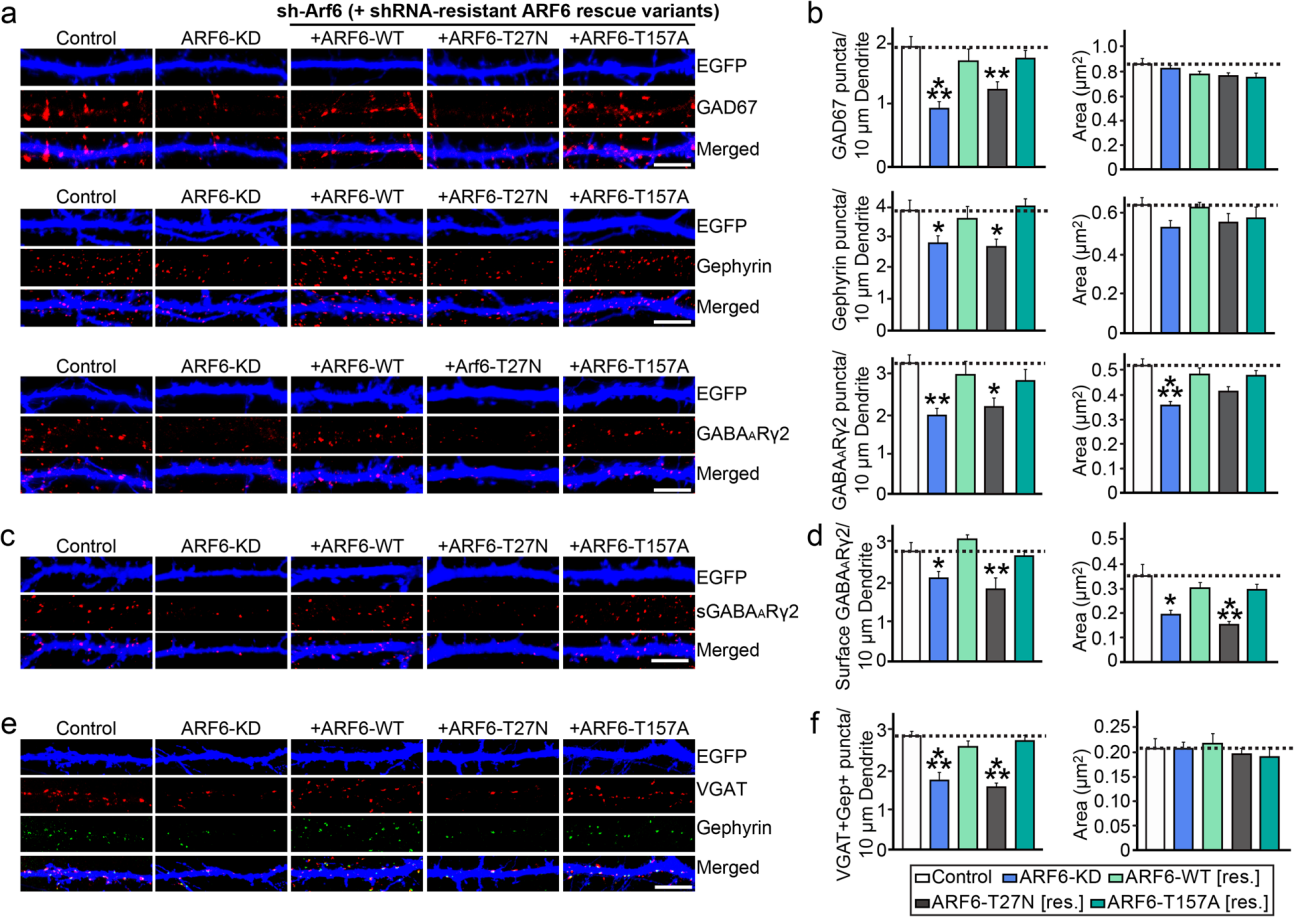
ARF6 activity is required for GABAergic synapse development in cultured neurons. **a** Cultured hippocampal neurons were transfected with a lentiviral vector expressing sh-Control, sh-Arf6, or coexpressing sh-Arf6 and shRNA-resistant ARF6 expression vectors (ARF6-WT, ARF6-T27 N, or ARF6-T157A) at DIV8 and analyzed at DIV14 by double-immunofluorescence staining with antibodies to EGFP (blue) and the indicated synaptic markers (GAD67, gephyrin, or GABA_A_Rγ2). **b** Summary data showing the effects of ARF6 KD on synaptic puncta density (left) and synaptic puncta size (right), measured using GAD67, gephyrin, and GABAARγ2 as synaptic markers. More than two dendrites per transfected neuron were analyzed and group-averaged. Data are presented as means ± SEMs from three independent experiments (*n* = 12–18 neurons; *p < 0.05, **p < 0.01, ***p < 0.001 vs. control; non-parametric ANOVA with Kruskal-Wallis test followed by post hoc Dunn’s multiple comparison test). **c** Cultured hippocampal neurons were transfected with a lentiviral vector expressing sh-Control, sh-Arf6, or coexpressing sh-Arf6 and shRNA-resistant ARF6 expression vectors (ARF6-WT, ARF6-T27 N, or ARF6-T157A) at DIV8 and analyzed at DIV14 by double-immunofluorescence staining with antibodies to EGFP (blue) and surface GABA_A_Rγ2 (red). **d** Summary data showing the effects of ARF6 KD on the density of surface GABAARγ2^+^ puncta (left) and size of surface GABA_A_Rγ2^+^ puncta (right). More than two dendrites per transfected neuron were analyzed and group-averaged. Data are presented as means ± SEMs from three independent experiments (*n* = 12–18 neurons; *p < 0.05, **p < 0.01, ***p < 0.001 vs. control; non-parametric ANOVA with Kruskal-Wallis test followed by post hoc Dunn’s multiple comparison test). **e** Cultured hippocampal neurons were transfected with a lentiviral vector expressing sh-Control, sh-Arf6, or coexpressing sh-Arf6 and shRNA-resistant ARF6 expression vectors (ARF6-WT, ARF6-T27 N, or ARF6-T157A) at DIV8 and analyzed at DIV14 by triple-immunofluorescence staining with antibodies to EGFP (blue), VGAT (red) and gephyrin (green). **f** Summary data showing the effects of ARF6 KD on the colocalized puncta density of VGAT and gephyrin (left) and size of colocalized puncta (right). More than two dendrites per transfected neuron were analyzed and group-averaged. Data are presented as means ± SEMs from three independent experiments (*n* = 16 neurons; ***p < 0.001 vs. control; non-parametric ANOVA with Kruskal-Wallis test followed by post hoc Dunn’s multiple comparison test)

To determine whether ARF6 impacts GABAergic synapse development, we first generated shRNA lentiviral vectors targeting ARF1 and ARF6 and confirmed their efficacy (Fig. 2a–d). Quantitative reverse transcription-polymerase chain reaction (qRT-PCR) showed that ARF1 and ARF6 mRNA levels were decreased by ~ 85% and ~ 90%, respectively, in cultured rat cortical neurons infected with the corresponding shRNA-expressing lentiviruses (Fig. 2b). In addition, semi-quantitative immunoblotting showed that shRNA targeting ARF6 decreased endogenous ARF6 protein levels (Fig. 2c, d). We then transfected cultured hippocampal neurons at DIV8 with validated shRNA lentiviral vectors targeting Arf1 (sh-Arf1), Arf6 (sh-Arf6) or EGFP only (sh-Control), and immunostained transfected neurons at DIV14 for the excitatory presynaptic marker VGLUT1, the excitatory postsynaptic marker PSD-95 (post-synaptic density protein 95), the inhibitory presynaptic marker GAD67, and the inhibitory postsynaptic markers, gephyrin and GABA_A_Rγ2 (Fig. 2e–g). As previously reported [3], knockdown of ARF1 (ARF1 KD) or ARF6 (ARF6 KD) significantly reduced the density of PSD-95^+^ and/or and VGLUT1^+^ puncta (Fig. 2e–g). Notably double-KD of ARF1 and ARF6 (ARF1/6 DKD) did not further decrease excitatory synaptic density compared with KD of either protein alone, suggesting that both ARF1 and ARF6 share common pathways in the maintenance of excitatory synapse structure in hippocampal neurons (Fig. 2e–g). Intriguingly, ARF6 KD also reduced the density of puncta positive for GAD67, gephyrin, or GABA_A_Rγ2; in contrast, ARF1 KD did not affect GABAergic synaptic puncta density (Fig. 2e–g). To investigate whether the modulation of inhibitory synaptic density by ARF6 requires ARF activity, we transfected cultured neurons at DIV8 with a lentiviral expression vector for EGFP only (shControl), ARF6-shRNA, or ARF6-shRNA and an shRNA resistant full-length ARF6 expression vector, and immunostained transfected neurons at DIV14 for various GABAergic synaptic markers. We found that the ARF6 KD-induced reduction in GABAergic synaptic puncta density, monitored by either a single synaptic marker (GAD67 or gephyrin) or both pre- and postsynaptic markers (VGAT and gephyrin), was completely rescued by coexpression of shRNA-resistant ARF6-WT or ARF6-T157A (a fast-recycling mutant), but not by coexpression of ARF-T27 N (a GTP-binding–defective mutant; Fig. 3a–f) [3]. In addition, reduced surface levels of GABA_A_Rγ2, a critical component of the synaptic GABA_A_ receptor, by ARF6 KD was normalized by coexpression of shRNA-resistant ARF6-WT or ARF6-T157A (Fig. 3c–d). Notably, expression of ARF6-Q67L (a GTP hydrolysis-resistant mutant) in either cultured hippocampal neurons or the DG of juvenile mice drastically altered neuronal morphology, precluding further analyses (data not shown; see also [3]). These results suggest that normal GTP-GDP cycling of ARF6 is critical for normal operation of GABAergic synapses.

### ARF6 is required for GABAergic synapse development in vivo

**Fig. 4:**
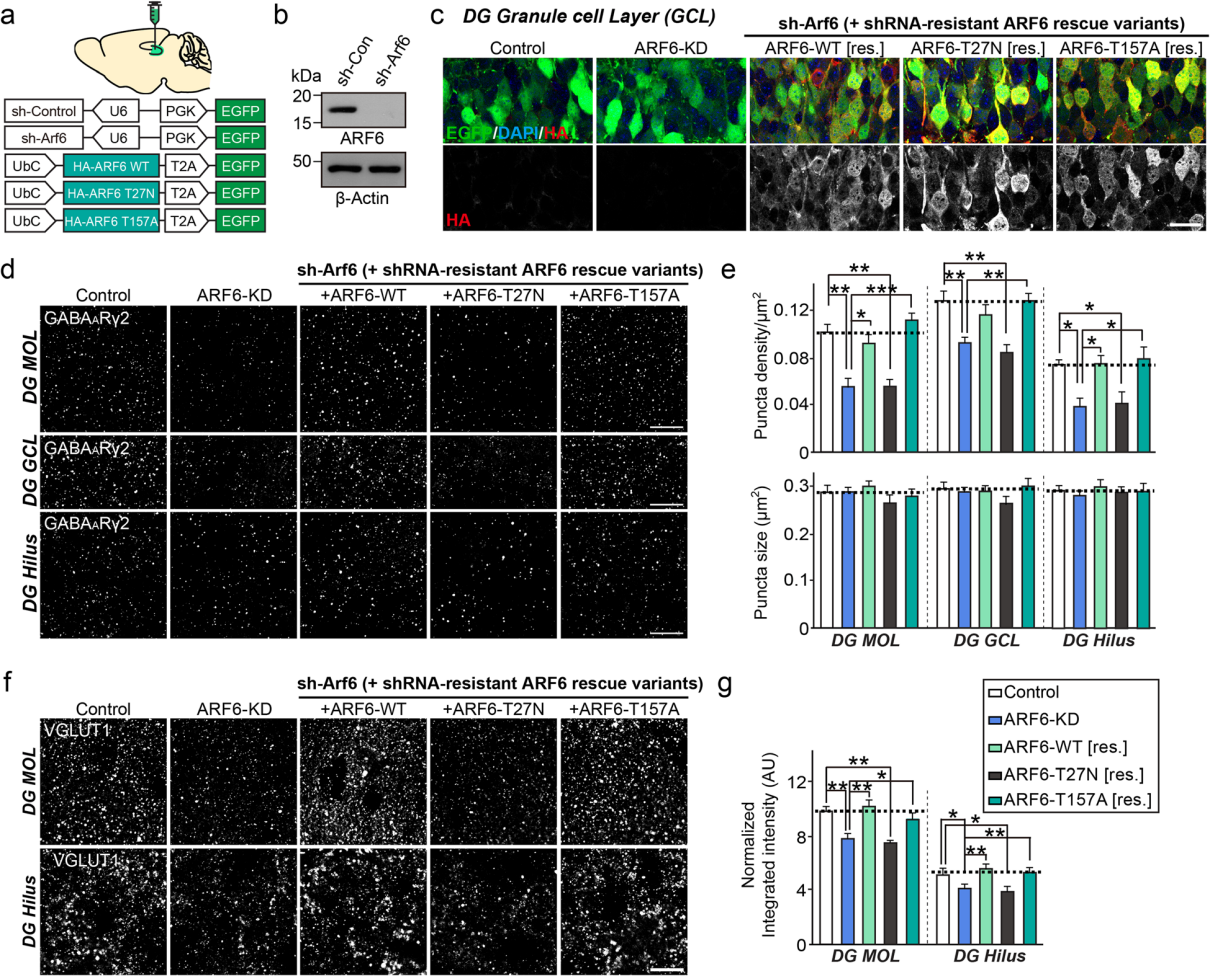
ARF6 activity is required for GABAergic synapse development in vivo*.* **a** Schematic diagram of AAV vectors expressing sh-Arf6 and HA-tagged ARF6 and its mutants (T27 N and T157A) used in **c**–**g**. **b** Immunoblotting analyses with ARF6 antibodies showing the KD efficacy of sh-ARF6 in vivo. Lysates from mouse brain stereotactically injected with AAVs expressing sh-ARF6 were collected and immunoblotted with anti-ARF6 antibodies. Anti-β-actin antibodies were used as normalization controls. **c** Representative images illustrating EGFP expression after AAV injection into the hippocampal DG region. Brain sections were immunostained for EGFP (green) or HA (red) and counterstained with DAPI (blue). Scale bar: 20 μm (applies to all images). **d** Representative images showing GABA_A_Rγ2^+^ puncta in the DG of mice stereotactically injected with AAVs expressing Control or sh-Arf6, or coexpressing sh-Arf6 and the indicated ARF6 variants (ARF6-WT, ARF6-T27 N, or ARF6-T157A). Scale bar, 20 μm (applies to all images). Abbreviations: MOL, molecular layer; GCL, granule cell layer. **e** Quantification of the density and size of GABA_A_Rγ2^+^ puncta per tissue area. Data are presented as means ± SEMs (*n* = 20–25 sections/4–5 mice; *p < 0.05, **p < 0.01, ***p < 0.001 vs. control; non-parametric ANOVA with Kruskal-Wallis test followed by post hoc Dunn’s multiple comparison test). **f** Representative images of AAV-infected neurons in DG molecular and hilar regions immunostained for the excitatory marker VGLUT1. Scale bar: 20 μm (applies to all images). **g** Quantification of VGLUT1^+^ puncta intensity per tissue area. Data are presented as means ± SEMs from 3 to 5 independent experiments (*n* = 22–30 sections/4–6 mice; *p < 0.05, **p < 0.01 vs. control; non-parametric ANOVA with Kruskal-Wallis test, followed by post hoc Dunn’s multiple comparison test)

To extend these observations in neurons in vivo, we used mice stereotactically injected with AAVs that express either sh-Arf6 (ARF6 KD) or sh-Control (Control) in the hippocampal DG and performed immunohistochemical analyses to probe whether ARF6 KD also influences structural aspects of GABAergic synapse development (Fig. 4a). ARF6 KD efficiency and shRNA-resistant ARF6 rescue vectors were validated by Western blotting with ARF6 antibodies and immunofluorescence analysis with HA antibodies, respectively (Fig. 4b, c). Quantitative immunofluorescence analyses revealed a significant decrease in the puncta intensity of the GABAergic synaptic marker GABA_A_Rγ2 in the DG granular cell layer and DG hilus and molecular layers (Fig. 4d, e). These changes in GABA_A_Rγ2 intensity in the DG of ARF6-KD mice were completely rescued by coexpression of shRNA-resistant ARF6-WT or ARF6-T157A, but not by coexpression of shRNA-resistant ARF6-T27 N (Fig. 4d, e). In keeping with previous observations, quantitative immunofluorescence analyses of the excitatory synaptic marker VGLUT1 revealed a reduction in the density of VGLUT1^+^ puncta in the DG molecular layer and hilus (Fig. 4f, g). Collectively, these data suggest that ARF6 is also required for GABAergic synapse development, similar to its established action at glutamatergic synapses.

### Loss of ARF6 accelerates seizure susceptibility in an ARF activity–dependent manner

**Fig. 5:**
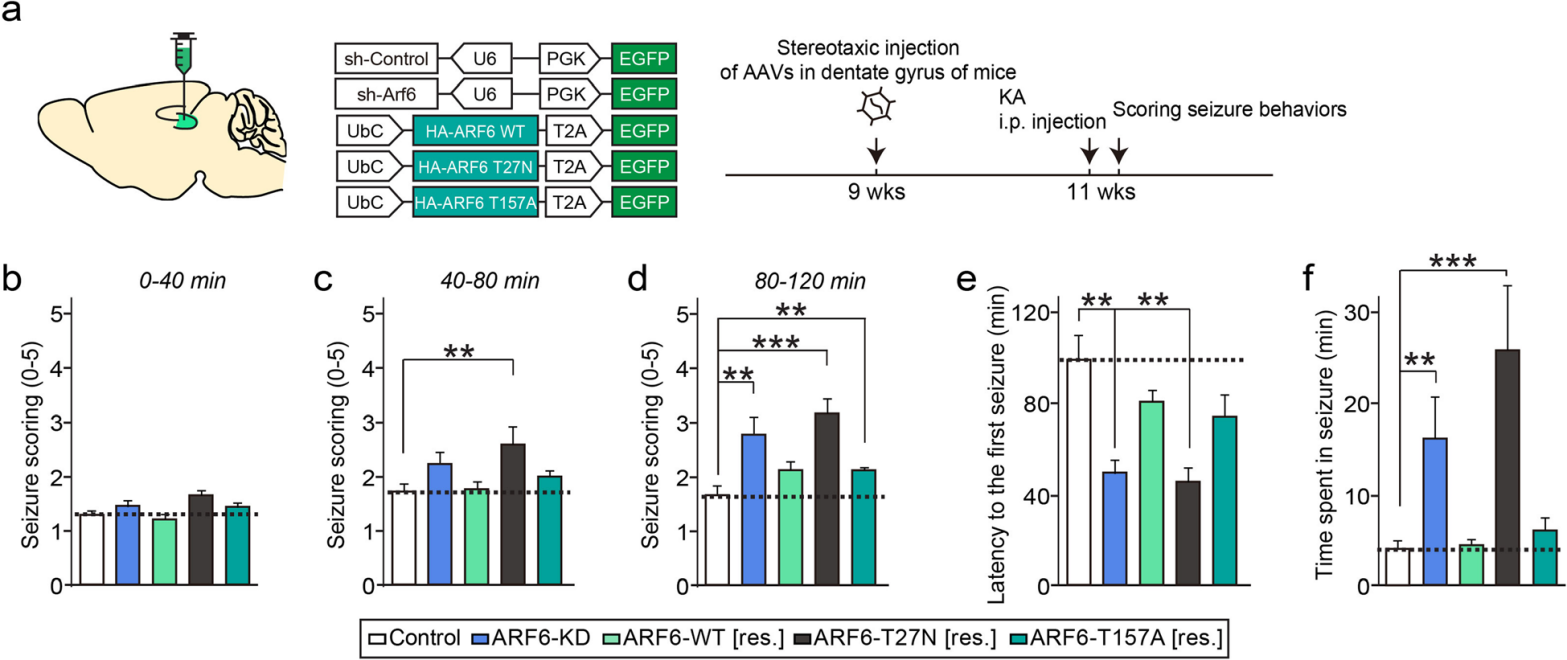
ARF6-KD mice exhibit a delayed, but significant, increase in seizure susceptibility. **a** Experimental scheme for seizure scoring. The DG region of the hippocampus of ~ 9-week-old WT mice was bilaterally injected with empty AAVs (Control) or ARF6-KD AAVs (ARF6 KD), or co-injected with the following: ARF6-KD AAVs and ARF6-WT–expressing AAVs (ARF6-WT [res.]); ARF6-KD AAVs and ARF6-T27 N–expressing AAVs (ARF6-T27 N [res.]); or ARF6-KD AAVs and ARF6-T157A–expressing AAVs (ARF6-T157A [res.]). Mice were intraperitoneally administered KA 2 weeks after AAV injections, and then analyzed by scoring seizures. **b**–**d** Quantification of mean score values for the first 40 min (**b**), second 40 min (**c**) and third 40 min (**d**) under each experimental condition (*n* = 9 mice/condition; **p < 0.01, ***p < 0.001 vs. control; Kruskal-Wallis test followed by Dunn’s post hoc test). **e** Quantification of latency to the first seizure after KA administration under each condition (n = 9 mice/condition; **p < 0.01, Kruskal-Wallis test followed by Dunn’s post hoc test). **f** Quantification of time spent in seizure under each condition (n = 9 mice/condition; **p < 0.01, ***p < 0.001 vs. control; Kruskal-Wallis test followed by Dunn’s post hoc test)

We next sought to determine whether loss of ARF6 induces network dysfunctions, which are often associated with impaired GABAergic synapse formation and function and a resulting imbalance in excitation/inhibition (E/I) ratio at synaptic and circuit levels [30, 35]. To test the effect of ARF6 KD on seizure susceptibility, we employed an acute kainic acid (KA)-induced epileptic mouse model, which has been extensively used to dissect molecular mechanisms underlying initial epileptogenesis event(s) that transforms normal neural networks into hypersynchronous networks. After stereotactic injection of a series of AAV vectors expressing ARF6 WT and its mutant variants (T27 N and T157A) [3] into the DG of ARF6-deficient mice, mice were intraperitoneally administered KA (20 mg/kg) and their seizure behaviors were scored (Fig. 5a). The severity of KA-induced convulsive seizures was assessed by scoring responses on a scale from 0 (no abnormal behavior) to 5 (death) using a revised Racine’s scale. Average seizure scores for the first 40 min after KA administration were comparable in ARF6-KD mice (1.41 ± 0.10) and control mice (1.33 ± 0.08) (Fig. 5b); average seizure scores for the next 40 min were 2.24 ± 0.18 and 1.75 ± 0.11 in ARF6-KD and control mice, respectively, indicating that the severity of seizure behaviors persisted in these mice (Fig. 5c), and average seizure scores for the last 40 min were ~ 1.6 fold higher in ARF6-KD mice than in control mice (Fig. 5d). Importantly, the increased seizure susceptibility observed in ARF6-KD mice was normalized by coexpression of shRNA-resistant ARF6 WT (2.15 ± 0.15 for the last 40 min) or ARF6-T157A (2.12 ± 0.07 for the last 40 min), but not by coexpression of shRNA-resistant ARF6-T27 N (2.58 ± 0.30 for the second 40 min and 3.14 ± 0.26 for last 40 min) (Fig. 5c, d). ARF6 KD decreased seizure latency, in association with an increase in the total time spent in seizures, both of which were normalized by expression of shRNA-resistant ARF6 WT and ARF6-T157A, but not shRNA-resistant ARF6-T27 N (Fig. 5e, f).

## Discussion

Molecular components of synapses have been identified, mostly by mass spectrometry analyses [36, 37]. Functional categorization of these proteins has revealed a number of GEFs and GAPs for small GTPases and shown that they constitute roughly ~ 10% of postsynaptic density proteins. Although many of these regulators have been studied at glutamatergic synapses, their roles at GABAergic synapses remain largely undefined. Recent efforts to identify GABAergic synaptic components and related molecular mechanisms have contributed to our understanding of how neural circuits are functionally balanced. However, even whether small GTPases and their regulators are expressed at GABAergic synapses has not been analyzed. In this study, we provide evidence that a fraction of ARF6 protein is localized to GABAergic synapses and functions to regulate GABAergic synapse number and hippocampal network activity. We demonstrated that an ARF6 deficiency leads to impaired GABAergic synapse development in an ARF6 activity-dependent manner in both cultured neurons and in vivo. In addition, the resultant GABAergic synaptic defect induced by ARF6 KD in the hippocampal DG area led to increased seizure susceptibility in mice, possibly owing to disinhibition of network activity in the hippocampal DG.

Strikingly, although the current study clearly showed that effects of ARF6 KD impact both glutamatergic and GABAergic synapse development in both hippocampal cultured neurons and mouse hippocampal DG region (Figs. 3 and 4), we speculate that phenotypic manifestations of ARF6 KD-triggered synapse loss are more prominent at GABAergic synapses, as shown by increased seizure susceptibility in ARF6-KD mice. Moreover, ARF1 KD specifically reduced the density of glutamatergic, but not GABAergic, synapses in cultured hippocampal neurons, suggesting that different small GTPases may participate in the development of distinct synapse types. Importantly, single KD of ARF1 or ARF6 decreased excitatory synapse density, whereas double KD of ARF1 and ARF6 had no further deleterious effect (Fig. 2), suggesting that ARF1 and ARF6 converge on the same downstream signaling cascades to regulate excitatory synapse development.

Similar to the mechanistic action of ARF6 at glutamatergic synapses, our study clearly demonstrated that active conversion of GDP-bound to GTP-bound states, but not the rate of conversion *per se*, are required for the action of ARF6 at GABAergic synapses (Fig. 3). In this regard, regulators of ARF6 activity, such as IQSEC3 (as a GEF) and GIT1 (as a GAP), act together. However, our observations suggest that ARF6 is not concentrated at synaptic sites (Fig. 1), whereas these regulators exhibit a relatively higher degree of localization at GABAergic synaptic sites [23, 35]. Thus, it is likely that these regulators also perform ARF6-independent functions.

Proper neuronal and network functions rely on balanced excitation and inhibition at diverse levels. Imbalances in the E/I ratio are responsible for the onset and/or progression of various neurological disorders, including epilepsy [28]. Thus, perturbation of ARF6-mediated GABAergic synapse development also contributes to defects in synaptic and circuit inhibition and the concomitant increase in the occurrence of epileptic seizures (Fig. 5). This idea is also supported by our molecular replacement experiments using various ARF6 variants, which showed that ARF6-T27 N failed to rescue ARF6-KD–induced epileptic phenotypes in mice.

Future studies should further dissect the detailed mechanisms by which ARF6 regulates various aspects of GABAergic synapse development. An intriguing possibility is that ARF6 directly regulates the exocytosis/endocytosis of GABA_A_ receptors. This idea is reminiscent of documented roles of ARF6 regulators (e.g. IQSEC1 and IQSEC2) at excitatory synapses, where IQSEC1 and IQSEC2 promote endocytosis of AMPA receptors [18, 19, 38]. However, epileptic-like behaviors observed in ARF6-KD mice could not be solely attributed to disruption of ARF6-mediated GABAergic synapse signaling, considering the well-documented roles of ARF proteins at glutamatergic synapses. It remains to be determined whether ARF6 differentially acts at specific synapse types and specific neurons. In addition, whether other ARFs besides ARF1 and ARF6 also perform similar or distinct actions at glutamatergic and GABAergic synapses should be investigated. Answering these issues will make an important contribution to our currently incomplete understanding of molecular organization at GABAergic synapses.

## Data Availability

The datasets generated and analyzed during the current study are available from the corresponding author upon reasonable request.

## Abbreviations

AAV: Adeno-associated virus
AMPA: α-amino-3-hydroxy-5-methyl-4-isoxazolepropionic acid
ARF: ADP-ribosylation factor
BRAG3: Brefeldin A-resistant Arf-GEF 3
DG: Dentate gyrus
GABA: Gamma-aminobutyric acid
GAPs: GTPase-activating proteins
GEFs: Guanine nucleotide exchange factors
IQSEC3: IQ motif and SEC7 domain-containing protein 3
KD: Knockdown
VGLUT1: Vesicular glutamate transporter 1
